# Supplementary material for: Analysis of Suicidal Behavior in Adult Inpatients with Anorexia Nervosa: Prevalence of Suicide Attempts and Non-Suicidal Self-Injury and Associated Factors—Data Before and After the COVID-19 Pandemic
Source: J Clin Med. 2024 Nov 18;13(22):6952. doi: 10.3390/jcm13226952 (PMC11594882; doi:10.3390/jcm13226952)
Supplement: Supplementary file 1 [file jcm-13-06952-s001.zip › jcm-3280326-supplementary.pdf]

Supplementary Materials

S1: Differences in psychometric scales between patients with and without SA.

	SA (N=)	no-SA (N =)	Statistical analysis	
	Mean (SD)	Mean (SD)	Z	p
Eating-related psychopathology				
EDI-2 drive for thinness	14.5 (7.6)	12.8 (7.7)	1.227	.220
EDI-2 bulimia	6.1 (5.7)	3.1 (4.9)	-3.025	**.002**
EDI-2 body dissatisfaction	16.8 (7.3)	14.6 (6.8)	-1.465	.143
EDI-2 ineffectiveness	16.5 (6.8)	11.7 (8.1)	-2.364	**.018**
EDI-2 perfectionism	5.7 (4)	5.7 (4.2)	-.074	.942
EDI-2 interpersonal distrust	9.4 (4.6)	7.2 (4.6)	-1.910	.056
EDI-2 interoceptive awareness	14.5 (8.1)	11.7 (11.5)	-1.568	.117
EDI-2 maturity fear	9.2 (6.8)	7.8 (5.4)	-.591	.555
EDI-2 asceticism	8.8 (5.7)	7.9 (5.1)	-.348	.728
EDI-2 impulsivity	9.3 (7.4)	6 (6.3)	-1.863	.062
EDI-2 social insecurity	11 (4.6)	8.9 (4.9)	-1.858	.063
EDE-Q restraint	3.9 (1.9)	3.3 (2.1)	-1.244	.214
EDE-Q eating concern	3.4 (1.4)	3.1 (1.7)	-1.193	.233
EDE-Q shape concern	4.9 (1.6)	4.1 (1.7)	-1.294	.212
EDE-Q weight concern	4.3 (1.7)	3.6 (1.8)	-2.002	**.045**
EDE-Q global score	4.1 (1.5)	3.5 (1.7)	-1.570	.116
General psychopathology				
BDI	21 (6.3)	16.3 (8.3)	-2.778	**.005**
STAI trait	65.8 (7.9)	57.8 (13.4)	-2.723	**.006**
STAI state	62.3 (10.2)	55.1 (13.7)	-2.419	**.016**

S2: S1: Differences in psychometric scales between patients with and without NSSI.

	SA (N=)	no-SA (N =)	Statistical analysis	
	Mean (SD)	Mean (SD)	Z	p
Eating-related psychopathology				
EDI-2 drive for thinness	17.2 (5.1)	12.3 (7.8)	-3.027	**.002**
EDI-2 bulimia	8 (6.4)	2.6 (4.4)	-5.742	**<.001**
EDI-2 body dissatisfaction	20.7 (5.9)	13.9 (6.5)	-5.000	**<.001**
EDI-2 ineffectiveness	18.9 (7.4)	11.1 (7.8)	-4.381	**<.001**
EDI-2 perfectionism	6.6 (4.5)	5.6 (4.1)	-1.001	.317
EDI-2 interpersonal distrust	9.2 (4.2)	7.1 (4.7)	-2.270	**.023**
EDI-2 interoceptive awareness	16.8 (6.3)	11.2 (11.6)	-3.580	**<.001**
EDI-2 maturity fear	9.6 (6.7)	7.7 (5.3)	-1.105	.269
EDI-2 asceticism	10.8 (4.9)	7.6 (5)	-3.018	**.003**
EDI-2 impulsivity	10.9 (6.8)	5.6 (6.1)	-3.926	**<.001**
EDI-2 social insecurity	12 (4.3)	8.6 (4.9)	-3.284	**.001**
EDE-Q restraint	4.7 (1.3)	3.2 (2.1)	-4.026	**<.001**
EDE-Q eating concern	4.2 (1.2)	2.9 (1.6)	-4.224	**<.001**
EDE-Q shape concern	5.2 (.8)	4 (1.7)	-3.865	**<.001**
EDE-Q weight concern	4.9 (1)	3.5 (1.8)	-4.018	**<.001**
EDE-Q global score			-4.340	**<.001**
General psychopathology				
BDI	22.6 (7.1)	15.9 (8)	-4.633	**<.001**
STAI trait	65.2 (9.4)	57.5 (13.4)	-3.305	**<.001**

STAI state	63.6 (9.3)	54.5 (13.8)	-3.544	**<.001**
